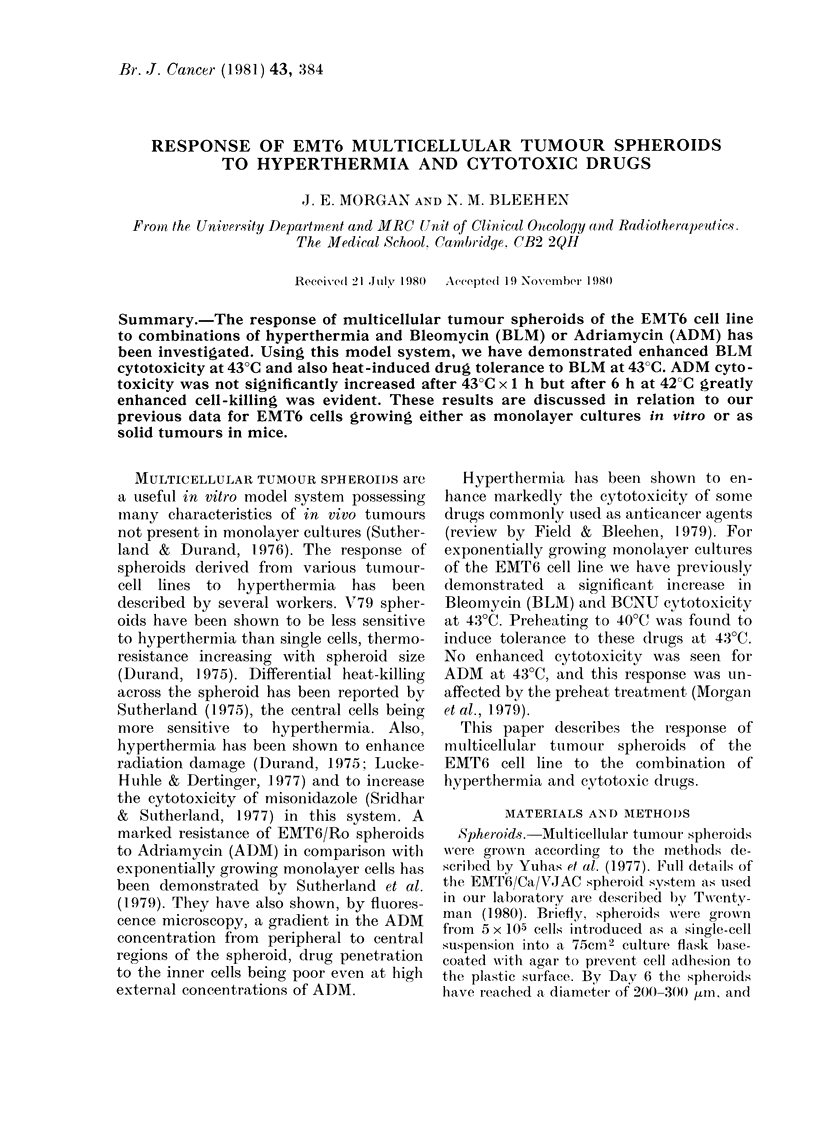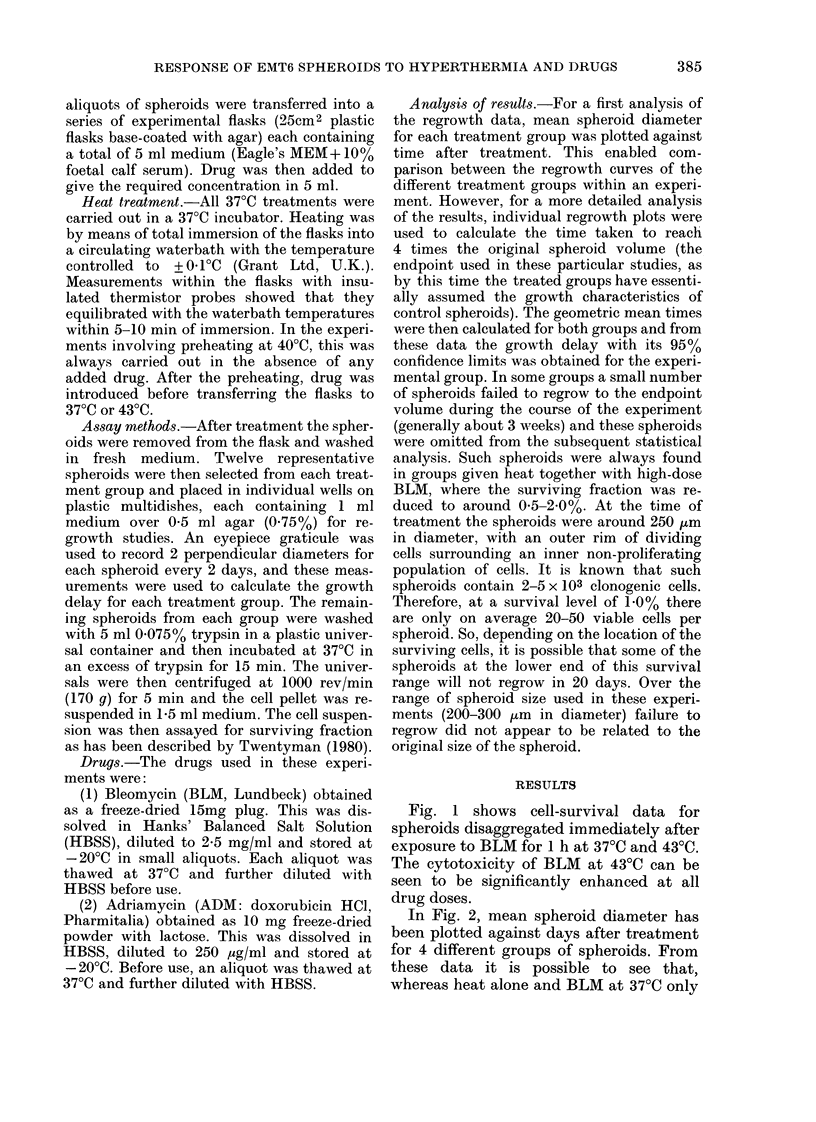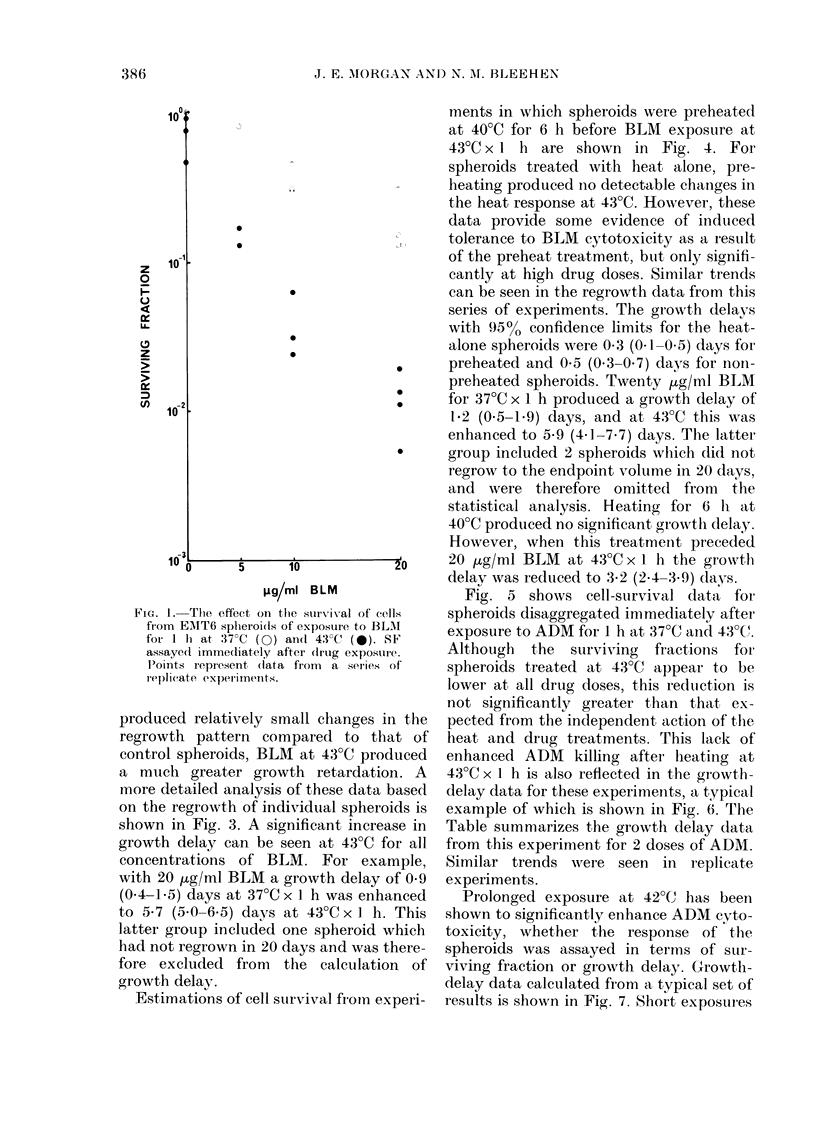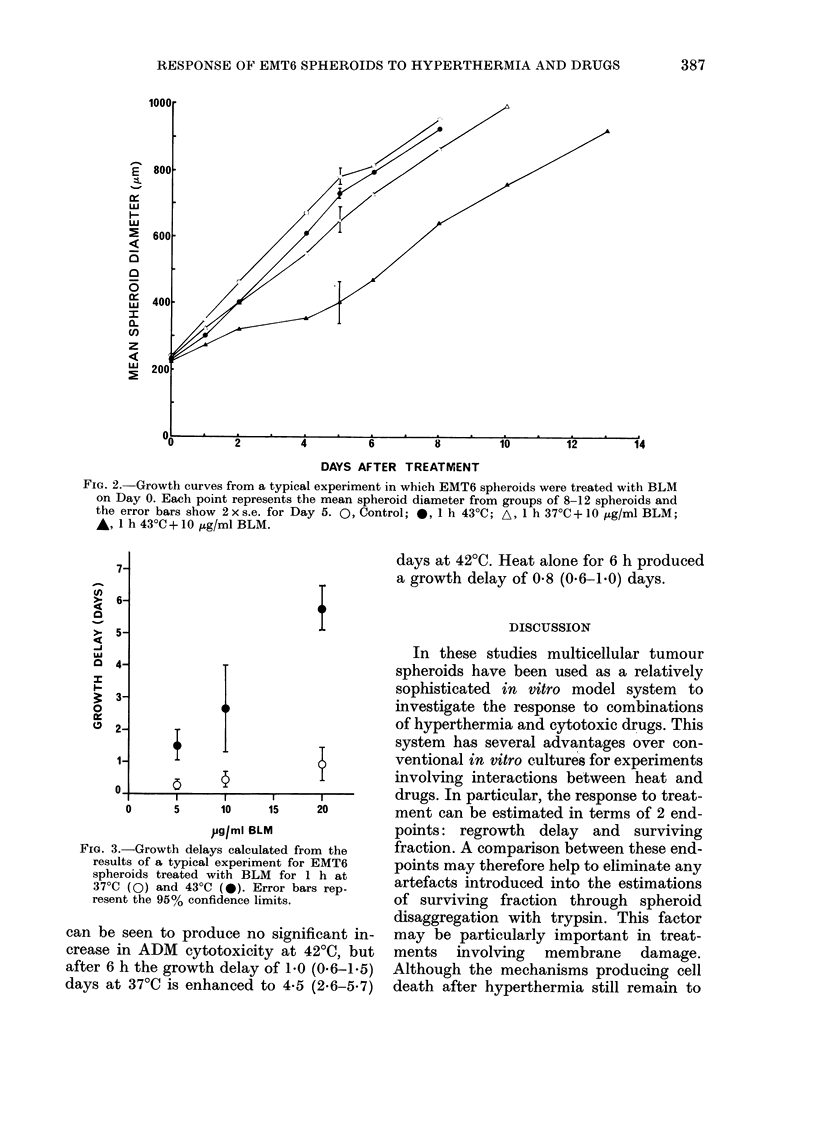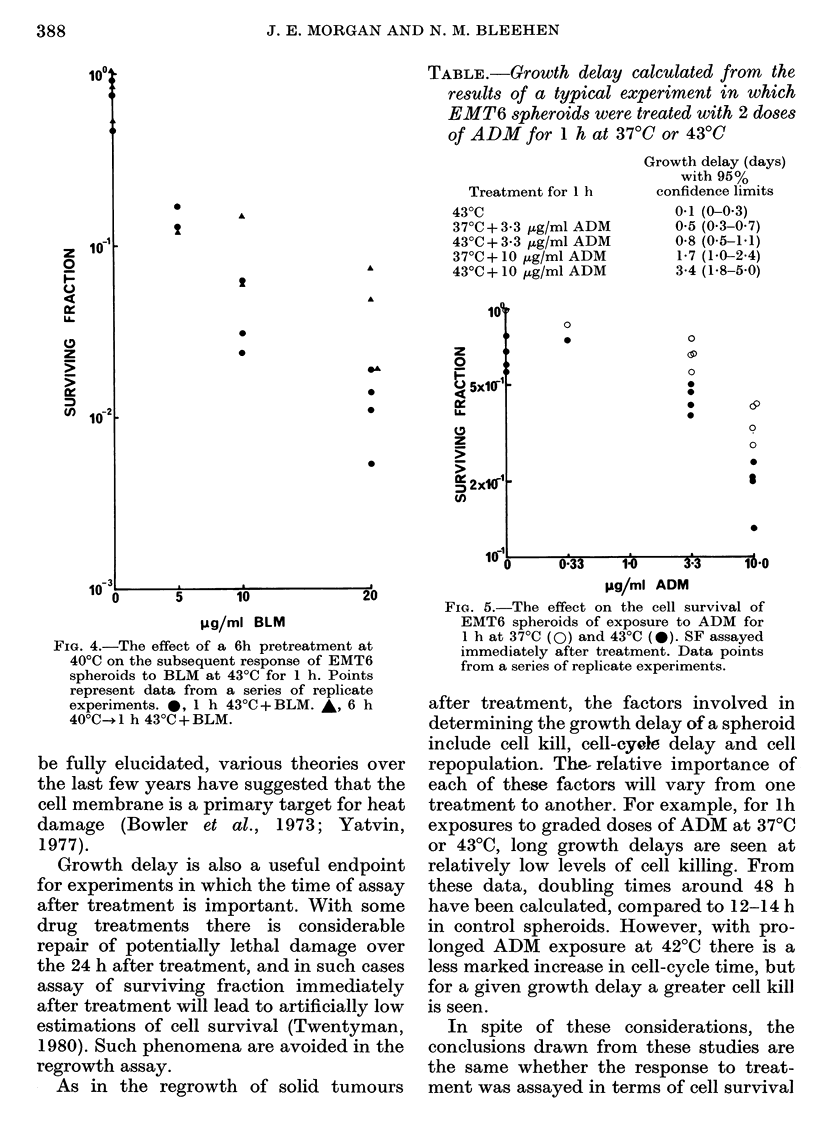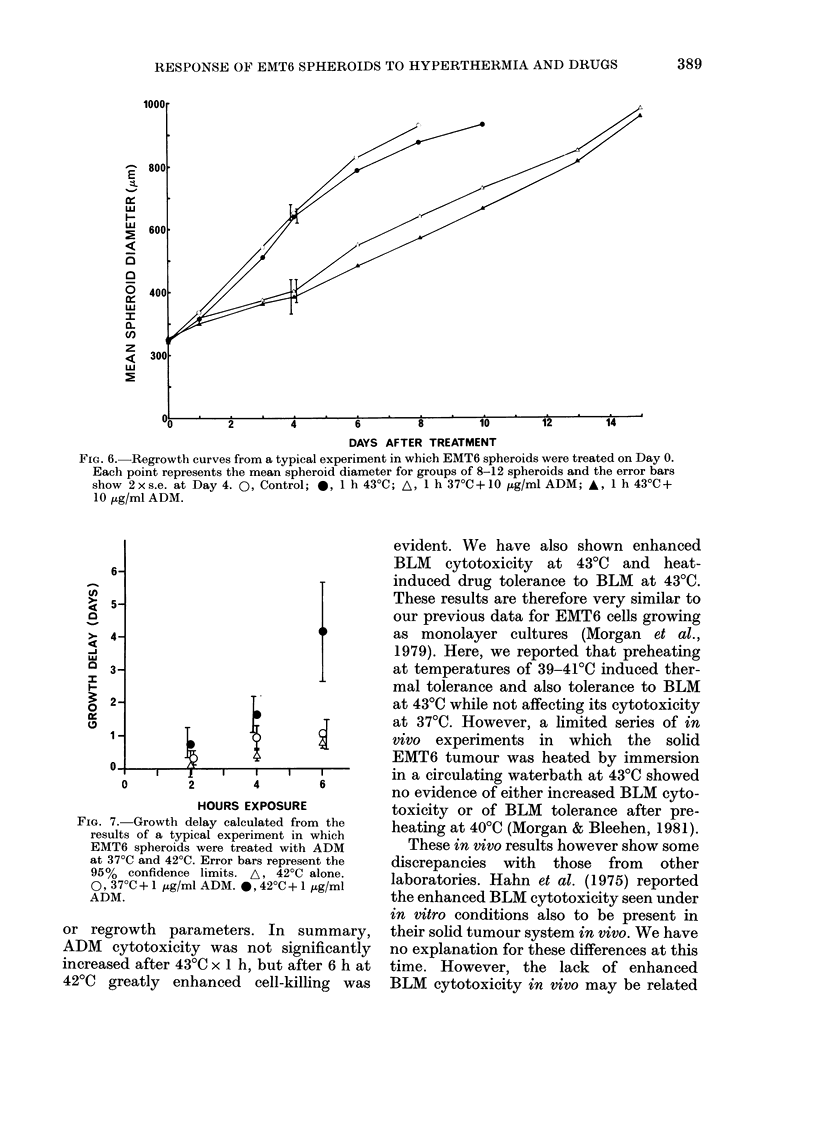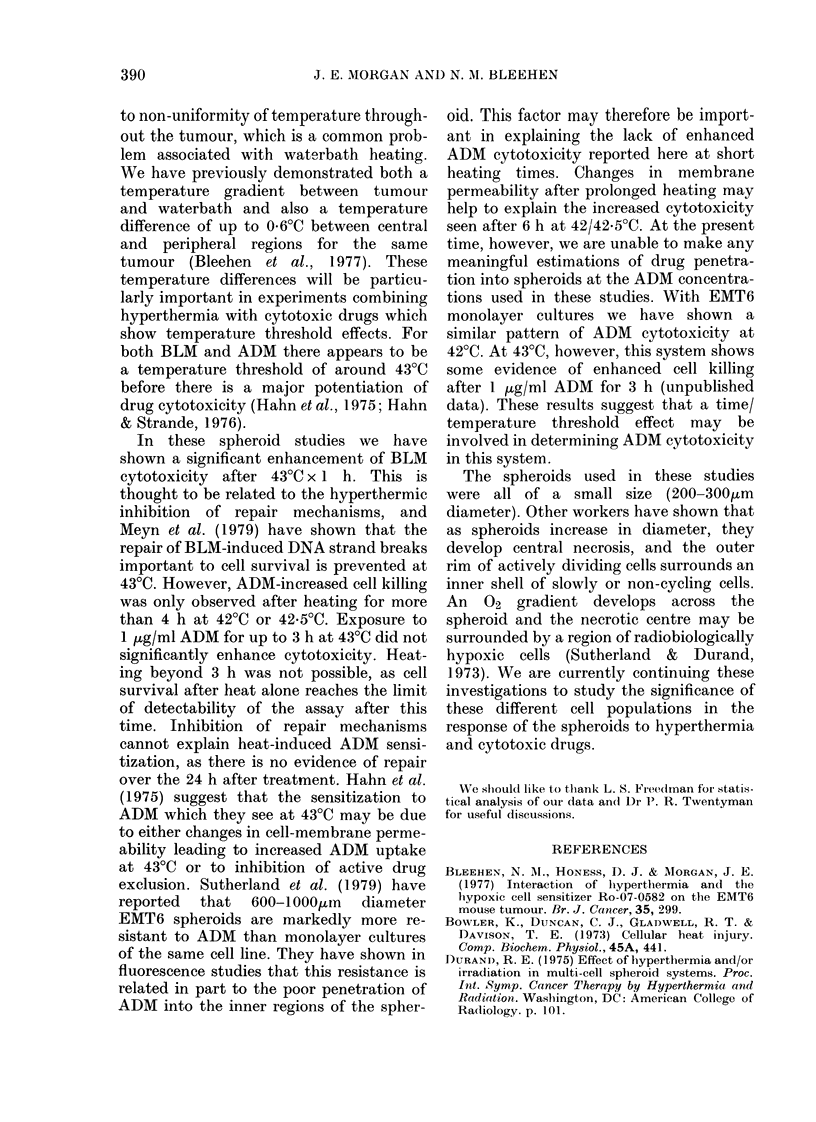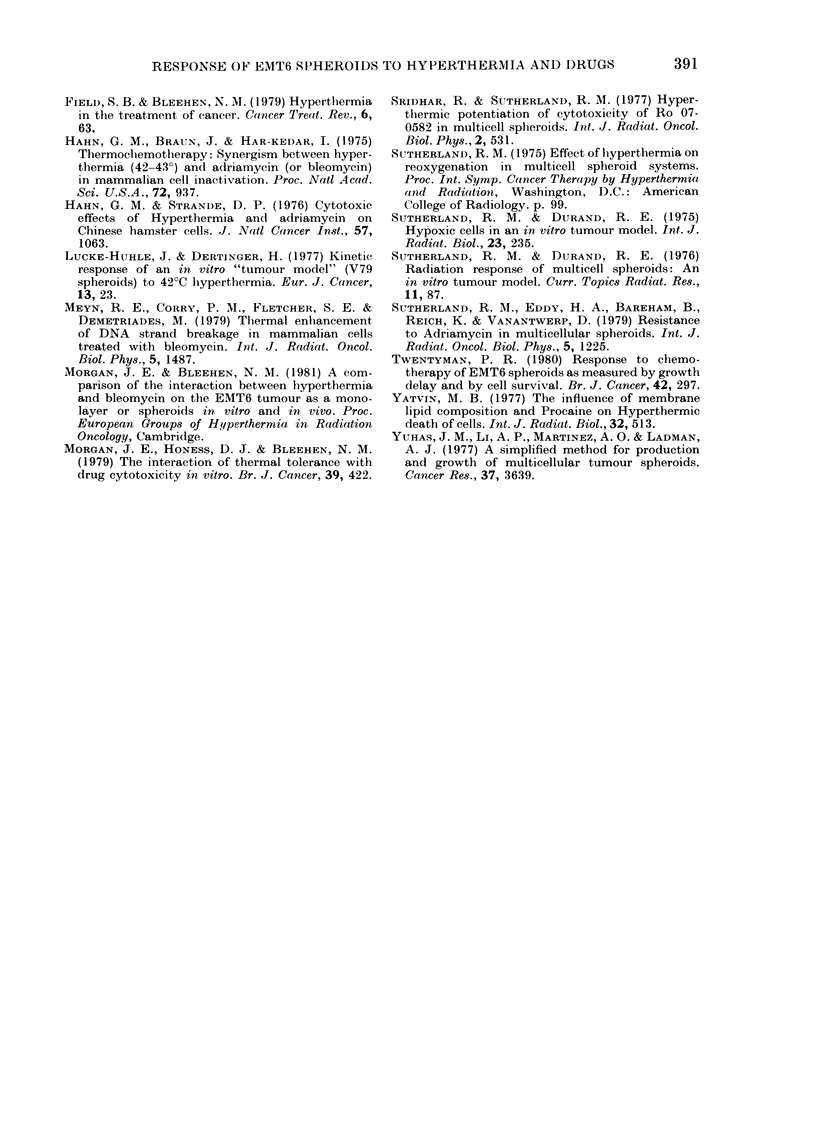# Response of EMT6 multicellular tumour spheroids to hyperthermia and cytotoxic drugs.

**DOI:** 10.1038/bjc.1981.59

**Published:** 1981-03

**Authors:** J. E. Morgan, N. M. Bleehen

## Abstract

The response of multicellular tumour spheroids of the EMT6 cell line to combinations of hyperthermia and Bleomycin (BLM) or Adriamycin (ADM) has been investigated. Using this model system, we have demonstrated enhanced BLM cytotoxicity at 43 degrees C and also heat-induced drug tolerance to BLM at 43 degrees C. ADM cytotoxicity was not significantly increased after 43 degrees C x 1 h but after 6 h at 42 degrees C greatly enhanced cell-killing was evident. These results are discussed in relation to our previous data for EMT6 cells growing either as monolayer cultures in vitro or as solid tumours in mice.


Br. J. Cancer (1981) 43, 384

### RESPONSE OF EMT6 MULTICELLULAR TUMOUR SPHEROIDS TO HYPERTHERMIA AND CYTOTOXIC DRUGS

J. E. MORGAN AND N. M. BLEEHEN

*From the University Department and MRC Unit of Clinical Oncology and Radiotherapeutics, The Medical School, Cambridge, CB2 2QH*

Received 21 July 1980 Accepted 19 November 1980

**Summary.—The response of multicellular tumour spheroids of the EMT6 cell line to combinations of hyperthermia and Bleomycin (BLM) or Adriamycin (ADM) has been investigated. Using this model system, we have demonstrated enhanced BLM cytotoxicity at 43°C and also heat-induced drug tolerance to BLM at 43°C. ADM cytotoxicity was not significantly increased after 43°C × 1 h but after 6 h at 42°C greatly enhanced cell-killing was evident. These results are discussed in relation to our previous data for EMT6 cells growing either as monolayer cultures *in vitro* or as solid tumours in mice.**

MULTICELLULAR TUMOUR SPHEROIDS are a useful *in vitro* model system possessing many characteristics of *in vivo* tumours not present in monolayer cultures (Sutherland & Durand, 1976). The response of spheroids derived from various tumour-cell lines to hyperthermia has been described by several workers. V79 spheroids have been shown to be less sensitive to hyperthermia than single cells, thermoresistance increasing with spheroid size (Durand, 1975). Differential heat-killing across the spheroid has been reported by Sutherland (1975), the central cells being more sensitive to hyperthermia. Also, hyperthermia has been shown to enhance radiation damage (Durand, 1975; Lucke-Huhle & Dertinger, 1977) and to increase the cytotoxicity of misonidazole (Sridhar & Sutherland, 1977) in this system. A marked resistance of EMT6/Ro spheroids to Adriamycin (ADM) in comparison with exponentially growing monolayer cells has been demonstrated by Sutherland *et al.* (1979). They have also shown, by fluorescence microscopy, a gradient in the ADM concentration from peripheral to central regions of the spheroid, drug penetration to the inner cells being poor even at high external concentrations of ADM.

Hyperthermia has been shown to enhance markedly the cytotoxicity of some drugs commonly used as anticancer agents (review by Field & Bleehen, 1979). For exponentially growing monolayer cultures of the EMT6 cell line we have previously demonstrated a significant increase in Bleomycin (BLM) and BCNU cytotoxicity at 43°C. Preheating to 40°C was found to induce tolerance to these drugs at 43°C. No enhanced cytotoxicity was seen for ADM at 43°C, and this response was unaffected by the preheat treatment (Morgan *et al.*, 1979).

This paper describes the response of multicellular tumour spheroids of the EMT6 cell line to the combination of hyperthermia and cytotoxic drugs.

### MATERIALS AND METHODS

*Spheroids.*—Multicellular tumour spheroids were grown according to the methods described by Yuhas *et al.* (1977). Full details of the EMT6/Ca/VJAC spheroid system as used in our laboratory are described by Twentyman (1980). Briefly, spheroids were grown from $5 \times 10^5$ cells introduced as a single-cell suspension into a $75 cm^2$ culture flask base-coated with agar to prevent cell adhesion to the plastic surface. By Day 6 the spheroids have reached a diameter of 200–300 $\mu m$, and aliquots of spheroids were transferred into a series of experimental flasks ($25cm^2$ plastic flasks base-coated with agar) each containing a total of 5 ml medium (Eagle's MEM+10% foetal calf serum). Drug was then added to give the required concentration in 5 ml.

*Heat treatment.*—All 37°C treatments were carried out in a 37°C incubator. Heating was by means of total immersion of the flasks into a circulating waterbath with the temperature controlled to ±0·1°C (Grant Ltd, U.K.). Measurements within the flasks with insulated thermistor probes showed that they equilibrated with the waterbath temperatures within 5–10 min of immersion. In the experiments involving preheating at 40°C, this was always carried out in the absence of any added drug. After the preheating, drug was introduced before transferring the flasks to 37°C or 43°C.

*Assay methods.*—After treatment the spheroids were removed from the flask and washed in fresh medium. Twelve representative spheroids were then selected from each treatment group and placed in individual wells on plastic multidishes, each containing 1 ml medium over 0·5 ml agar (0·75%) for regrowth studies. An eyepiece graticule was used to record 2 perpendicular diameters for each spheroid every 2 days, and these measurements were used to calculate the growth delay for each treatment group. The remaining spheroids from each group were washed with 5 ml 0·075% trypsin in a plastic universal container and then incubated at 37°C in an excess of trypsin for 15 min. The universals were then centrifuged at 1000 rev/min (170 *g*) for 5 min and the cell pellet was resuspended in 1·5 ml medium. The cell suspension was then assayed for surviving fraction as has been described by Twentyman (1980).

*Drugs.*—The drugs used in these experiments were:

(1) Bleomycin (BLM, Lundbeck) obtained as a freeze-dried 15mg plug. This was dissolved in Hanks' Balanced Salt Solution (HBSS), diluted to 2·5 mg/ml and stored at −20°C in small aliquots. Each aliquot was thawed at 37°C and further diluted with HBSS before use.

(2) Adriamycin (ADM: doxorubicin HCl, Pharmitalia) obtained as 10 mg freeze-dried powder with lactose. This was dissolved in HBSS, diluted to 250 μg/ml and stored at −20°C. Before use, an aliquot was thawed at 37°C and further diluted with HBSS.

*Analysis of results.*—For a first analysis of the regrowth data, mean spheroid diameter for each treatment group was plotted against time after treatment. This enabled comparison between the regrowth curves of the different treatment groups within an experiment. However, for a more detailed analysis of the results, individual regrowth plots were used to calculate the time taken to reach 4 times the original spheroid volume (the endpoint used in these particular studies, as by this time the treated groups have essentially assumed the growth characteristics of control spheroids). The geometric mean times were then calculated for both groups and from these data the growth delay with its 95% confidence limits was obtained for the experimental group. In some groups a small number of spheroids failed to regrow to the endpoint volume during the course of the experiment (generally about 3 weeks) and these spheroids were omitted from the subsequent statistical analysis. Such spheroids were always found in groups given heat together with high-dose BLM, where the surviving fraction was reduced to around 0·5–2·0%. At the time of treatment the spheroids were around 250 μm in diameter, with an outer rim of dividing cells surrounding an inner non-proliferating population of cells. It is known that such spheroids contain $2–5 \times 10^3$ clonogenic cells. Therefore, at a survival level of 1·0% there are only on average 20–50 viable cells per spheroid. So, depending on the location of the surviving cells, it is possible that some of the spheroids at the lower end of this survival range will not regrow in 20 days. Over the range of spheroid size used in these experiments (200–300 μm in diameter) failure to regrow did not appear to be related to the original size of the spheroid.

### RESULTS

Fig. 1 shows cell-survival data for spheroids disaggregated immediately after exposure to BLM for 1 h at 37°C and 43°C. The cytotoxicity of BLM at 43°C can be seen to be significantly enhanced at all drug doses.

In Fig. 2, mean spheroid diameter has been plotted against days after treatment for 4 different groups of spheroids. From these data it is possible to see that, whereas heat alone and BLM at 37°C only

FIG. 1.—The effect on the survival of cells from EMT6 spheroids of exposure to BLM for 1 h at 37°C (○) and 43°C (●). SF assayed immediately after drug exposure. Points represent data from a series of replicate experiments.

produced relatively small changes in the regrowth pattern compared to that of control spheroids, BLM at 43°C produced a much greater growth retardation. A more detailed analysis of these data based on the regrowth of individual spheroids is shown in Fig. 3. A significant increase in growth delay can be seen at 43°C for all concentrations of BLM. For example, with 20 $\mu$g/ml BLM a growth delay of 0·9 (0·4–1·5) days at 37°C × 1 h was enhanced to 5·7 (5·0–6·5) days at 43°C × 1 h. This latter group included one spheroid which had not regrown in 20 days and was therefore excluded from the calculation of growth delay.

Estimations of cell survival from experiments in which spheroids were preheated at 40°C for 6 h before BLM exposure at 43°C × 1 h are shown in Fig. 4. For spheroids treated with heat alone, preheating produced no detectable changes in the heat response at 43°C. However, these data provide some evidence of induced tolerance to BLM cytotoxicity as a result of the preheat treatment, but only significantly at high drug doses. Similar trends can be seen in the regrowth data from this series of experiments. The growth delays with 95% confidence limits for the heat-alone spheroids were 0·3 (0·1–0·5) days for preheated and 0·5 (0·3–0·7) days for non-preheated spheroids. Twenty $\mu$g/ml BLM for 37°C × 1 h produced a growth delay of 1·2 (0·5–1·9) days, and at 43°C this was enhanced to 5·9 (4·1–7·7) days. The latter group included 2 spheroids which did not regrow to the endpoint volume in 20 days, and were therefore omitted from the statistical analysis. Heating for 6 h at 40°C produced no significant growth delay. However, when this treatment preceded 20 $\mu$g/ml BLM at 43°C × 1 h the growth delay was reduced to 3·2 (2·4–3·9) days.

Fig. 5 shows cell-survival data for spheroids disaggregated immediately after exposure to ADM for 1 h at 37°C and 43°C. Although the surviving fractions for spheroids treated at 43°C appear to be lower at all drug doses, this reduction is not significantly greater than that expected from the independent action of the heat and drug treatments. This lack of enhanced ADM killing after heating at 43°C × 1 h is also reflected in the growth-delay data for these experiments, a typical example of which is shown in Fig. 6. The Table summarizes the growth delay data from this experiment for 2 doses of ADM. Similar trends were seen in replicate experiments.

Prolonged exposure at 42°C has been shown to significantly enhance ADM cytotoxicity, whether the response of the spheroids was assayed in terms of surviving fraction or growth delay. Growth-delay data calculated from a typical set of results is shown in Fig. 7. Short exposures

FIG. 2.—Growth curves from a typical experiment in which EMT6 spheroids were treated with BLM on Day 0. Each point represents the mean spheroid diameter from groups of 8–12 spheroids and the error bars show 2 × s.e. for Day 5. ○, Control; ●, 1 h 43°C; △, 1 h 37°C + 10 μg/ml BLM; ▲, 1 h 43°C + 10 μg/ml BLM.

FIG. 3.—Growth delays calculated from the results of a typical experiment for EMT6 spheroids treated with BLM for 1 h at 37°C (○) and 43°C (●). Error bars represent the 95% confidence limits.

can be seen to produce no significant increase in ADM cytotoxicity at 42°C, but after 6 h the growth delay of 1·0 (0·6–1·5) days at 37°C is enhanced to 4·5 (2·6–5·7) days at 42°C. Heat alone for 6 h produced a growth delay of 0·8 (0·6–1·0) days.

### DISCUSSION

In these studies multicellular tumour spheroids have been used as a relatively sophisticated *in vitro* model system to investigate the response to combinations of hyperthermia and cytotoxic drugs. This system has several advantages over conventional *in vitro* cultures for experiments involving interactions between heat and drugs. In particular, the response to treatment can be estimated in terms of 2 end-points: regrowth delay and surviving fraction. A comparison between these end-points may therefore help to eliminate any artefacts introduced into the estimations of surviving fraction through spheroid disaggregation with trypsin. This factor may be particularly important in treatments involving membrane damage. Although the mechanisms producing cell death after hyperthermia still remain to

FIG. 4.—The effect of a 6h pretreatment at 40°C on the subsequent response of EMT6 spheroids to BLM at 43°C for 1 h. Points represent data from a series of replicate experiments. ●, 1 h 43°C+BLM. ▲, 6 h 40°C→1 h 43°C+BLM.

TABLE.—*Growth delay calculated from the results of a typical experiment in which EMT6 spheroids were treated with* 2 *doses of ADM for* 1 *h at* 37°C *or* 43°C

| Treatment for 1 h | Growth delay (days) with 95% confidence limits |
|---|---|
| 43°C | 0·1 (0–0·3) |
| 37°C+3·3 μg/ml ADM | 0·5 (0·3–0·7) |
| 43°C+3·3 μg/ml ADM | 0·8 (0·5–1·1) |
| 37°C+10 μg/ml ADM | 1·7 (1·0–2·4) |
| 43°C+10 μg/ml ADM | 3·4 (1·8–5·0) |

FIG. 5.—The effect on the cell survival of EMT6 spheroids of exposure to ADM for 1 h at 37°C (○) and 43°C (●). SF assayed immediately after treatment. Data points from a series of replicate experiments.

be fully elucidated, various theories over the last few years have suggested that the cell membrane is a primary target for heat damage (Bowler *et al.*, 1973; Yatvin, 1977).

Growth delay is also a useful endpoint for experiments in which the time of assay after treatment is important. With some drug treatments there is considerable repair of potentially lethal damage over the 24 h after treatment, and in such cases assay of surviving fraction immediately after treatment will lead to artificially low estimations of cell survival (Twentyman, 1980). Such phenomena are avoided in the regrowth assay.

As in the regrowth of solid tumours after treatment, the factors involved in determining the growth delay of a spheroid include cell kill, cell-cycle delay and cell repopulation. The relative importance of each of these factors will vary from one treatment to another. For example, for 1h exposures to graded doses of ADM at 37°C or 43°C, long growth delays are seen at relatively low levels of cell killing. From these data, doubling times around 48 h have been calculated, compared to 12–14 h in control spheroids. However, with prolonged ADM exposure at 42°C there is a less marked increase in cell-cycle time, but for a given growth delay a greater cell kill is seen.

In spite of these considerations, the conclusions drawn from these studies are the same whether the response to treatment was assayed in terms of cell survival

FIG. 6.—Regrowth curves from a typical experiment in which EMT6 spheroids were treated on Day 0. Each point represents the mean spheroid diameter for groups of 8–12 spheroids and the error bars show 2 × s.e. at Day 4. ○, Control; ●, 1 h 43°C; △, 1 h 37°C + 10 μg/ml ADM; ▲, 1 h 43°C + 10 μg/ml ADM.

FIG. 7.—Growth delay calculated from the results of a typical experiment in which EMT6 spheroids were treated with ADM at 37°C and 42°C. Error bars represent the 95% confidence limits. △, 42°C alone. ○, 37°C + 1 μg/ml ADM. ●, 42°C + 1 μg/ml ADM.

or regrowth parameters. In summary, ADM cytotoxicity was not significantly increased after 43°C × 1 h, but after 6 h at 42°C greatly enhanced cell-killing was evident. We have also shown enhanced BLM cytotoxicity at 43°C and heat-induced drug tolerance to BLM at 43°C. These results are therefore very similar to our previous data for EMT6 cells growing as monolayer cultures (Morgan *et al.*, 1979). Here, we reported that preheating at temperatures of 39–41°C induced thermal tolerance and also tolerance to BLM at 43°C while not affecting its cytotoxicity at 37°C. However, a limited series of *in vivo* experiments in which the solid EMT6 tumour was heated by immersion in a circulating waterbath at 43°C showed no evidence of either increased BLM cytotoxicity or of BLM tolerance after preheating at 40°C (Morgan & Bleehen, 1981).

These *in vivo* results however show some discrepancies with those from other laboratories. Hahn *et al.* (1975) reported the enhanced BLM cytotoxicity seen under *in vitro* conditions also to be present in their solid tumour system *in vivo*. We have no explanation for these differences at this time. However, the lack of enhanced BLM cytotoxicity *in vivo* may be related to non-uniformity of temperature throughout the tumour, which is a common problem associated with waterbath heating. We have previously demonstrated both a temperature gradient between tumour and waterbath and also a temperature difference of up to 0·6°C between central and peripheral regions for the same tumour (Bleehen *et al.*, 1977). These temperature differences will be particularly important in experiments combining hyperthermia with cytotoxic drugs which show temperature threshold effects. For both BLM and ADM there appears to be a temperature threshold of around 43°C before there is a major potentiation of drug cytotoxicity (Hahn *et al.*, 1975; Hahn & Strande, 1976).

In these spheroid studies we have shown a significant enhancement of BLM cytotoxicity after 43°C × 1 h. This is thought to be related to the hyperthermic inhibition of repair mechanisms, and Meyn *et al.* (1979) have shown that the repair of BLM-induced DNA strand breaks important to cell survival is prevented at 43°C. However, ADM-increased cell killing was only observed after heating for more than 4 h at 42°C or 42·5°C. Exposure to 1 μg/ml ADM for up to 3 h at 43°C did not significantly enhance cytotoxicity. Heating beyond 3 h was not possible, as cell survival after heat alone reaches the limit of detectability of the assay after this time. Inhibition of repair mechanisms cannot explain heat-induced ADM sensitization, as there is no evidence of repair over the 24 h after treatment. Hahn *et al.* (1975) suggest that the sensitization to ADM which they see at 43°C may be due to either changes in cell-membrane permeability leading to increased ADM uptake at 43°C or to inhibition of active drug exclusion. Sutherland *et al.* (1979) have reported that 600–1000μm diameter EMT6 spheroids are markedly more resistant to ADM than monolayer cultures of the same cell line. They have shown in fluorescence studies that this resistance is related in part to the poor penetration of ADM into the inner regions of the spheroid. This factor may therefore be important in explaining the lack of enhanced ADM cytotoxicity reported here at short heating times. Changes in membrane permeability after prolonged heating may help to explain the increased cytotoxicity seen after 6 h at 42/42·5°C. At the present time, however, we are unable to make any meaningful estimations of drug penetration into spheroids at the ADM concentrations used in these studies. With EMT6 monolayer cultures we have shown a similar pattern of ADM cytotoxicity at 42°C. At 43°C, however, this system shows some evidence of enhanced cell killing after 1 μg/ml ADM for 3 h (unpublished data). These results suggest that a time/temperature threshold effect may be involved in determining ADM cytotoxicity in this system.

The spheroids used in these studies were all of a small size (200–300μm diameter). Other workers have shown that as spheroids increase in diameter, they develop central necrosis, and the outer rim of actively dividing cells surrounds an inner shell of slowly or non-cycling cells. An $O_2$ gradient develops across the spheroid and the necrotic centre may be surrounded by a region of radiobiologically hypoxic cells (Sutherland & Durand, 1973). We are currently continuing these investigations to study the significance of these different cell populations in the response of the spheroids to hyperthermia and cytotoxic drugs.

We should like to thank L. S. Freedman for statistical analysis of our data and Dr P. R. Twentyman for useful discussions.

### REFERENCES

Bleehen, N. M., Honess, D. J. & Morgan, J. E. (1977) Interaction of hyperthermia and the hypoxic cell sensitizer Ro-07-0582 on the EMT6 mouse tumour. *Br. J. Cancer*, **35**, 299.

Bowler, K., Duncan, C. J., Gladwell, R. T. & Davison, T. E. (1973) Cellular heat injury. *Comp. Biochem. Physiol.*, **45A**, 441.

Durand, R. E. (1975) Effect of hyperthermia and/or irradiation in multi-cell spheroid systems. *Proc. Int. Symp. Cancer Therapy by Hyperthermia and Radiation.* Washington, DC: American College of Radiology. p. 101.

FIELD, S. B. & BLEEHEN, N. M. (1979) Hyperthermia in the treatment of cancer. *Cancer Treat. Rev.*, **6**, 63.

HAHN, G. M., BRAUN, J. & HAR-KEDAR, I. (1975) Thermochemotherapy: Synergism between hyperthermia (42–43°) and adriamycin (or bleomycin) in mammalian cell inactivation. *Proc. Natl Acad. Sci. U.S.A.*, **72**, 937.

HAHN, G. M. & STRANDE, D. P. (1976) Cytotoxic effects of Hyperthermia and adriamycin on Chinese hamster cells. *J. Natl Cancer Inst.*, **57**, 1063.

LUCKE-HUHLE, J. & DERTINGER, H. (1977) Kinetic response of an *in vitro* "tumour model" (V79 spheroids) to 42°C hyperthermia. *Eur. J. Cancer*, **13**, 23.

MEYN, R. E., CORRY, P. M., FLETCHER, S. E. & DEMETRIADES, M. (1979) Thermal enhancement of DNA strand breakage in mammalian cells treated with bleomycin. *Int. J. Radiat. Oncol. Biol. Phys.*, **5**, 1487.

MORGAN, J. E. & BLEEHEN, N. M. (1981) A comparison of the interaction between hyperthermia and bleomycin on the EMT6 tumour as a monolayer or spheroids *in vitro* and *in vivo*. *Proc. European Groups of Hyperthermia in Radiation Oncology*, Cambridge.

MORGAN, J. E., HONESS, D. J. & BLEEHEN, N. M. (1979) The interaction of thermal tolerance with drug cytotoxicity *in vitro*. *Br. J. Cancer*, **39**, 422.

SRIDHAR, R. & SUTHERLAND, R. M. (1977) Hyperthermic potentiation of cytotoxicity of Ro 07-0582 in multicell spheroids. *Int. J. Radiat. Oncol. Biol. Phys.*, **2**, 531.

SUTHERLAND, R. M. (1975) Effect of hyperthermia on reoxygenation in multicell spheroid systems. *Proc. Int. Symp. Cancer Therapy by Hyperthermia and Radiation*, Washington, D.C.: American College of Radiology. p. 99.

SUTHERLAND, R. M. & DURAND, R. E. (1975) Hypoxic cells in an *in vitro* tumour model. *Int. J. Radiat. Biol.*, **23**, 235.

SUTHERLAND, R. M. & DURAND, R. E. (1976) Radiation response of multicell spheroids: An *in vitro* tumour model. *Curr. Topics Radiat. Res.*, **11**, 87.

SUTHERLAND, R. M., EDDY, H. A., BAREHAM, B., REICH, K. & VANANTWERP, D. (1979) Resistance to Adriamycin in multicellular spheroids. *Int. J. Radiat. Oncol. Biol. Phys.*, **5**, 1225.

TWENTYMAN, P. R. (1980) Response to chemotherapy of EMT6 spheroids as measured by growth delay and by cell survival. *Br. J. Cancer*, **42**, 297.

YATVIN, M. B. (1977) The influence of membrane lipid composition and Procaine on Hyperthermic death of cells. *Int. J. Radiat. Biol.*, **32**, 513.

YUHAS, J. M., LI, A. P., MARTINEZ, A. O. & LADMAN, A. J. (1977) A simplified method for production and growth of multicellular tumour spheroids. *Cancer Res.*, **37**, 3639.